# Tau-Mediated Dysregulation of Neuroplasticity and Glial Plasticity

**DOI:** 10.3389/fnmol.2020.00151

**Published:** 2020-08-21

**Authors:** Emily J. Koller, Paramita Chakrabarty

**Affiliations:** ^1^Department of Neuroscience, University of Florida, Gainesville, FL, United States; ^2^Center for Translational Research in Neurodegenerative Disease, University of Florida, Gainesville, FL, United States; ^3^McKnight Brain Institute, University of Florida, Gainesville, FL, United States

**Keywords:** synaptic plasticity, glial plasticity, Alzheimer’s disease, tauopathy, therapeutics

## Abstract

The inability of individual neurons to compensate for aging-related damage leads to a gradual loss of functional plasticity in the brain accompanied by progressive impairment in learning and memory. Whereas this loss in neuroplasticity is gradual during normal aging, in neurodegenerative diseases such as Alzheimer’s disease (AD), this loss is accelerated dramatically, leading to the incapacitation of patients within a decade of onset of cognitive symptoms. The mechanisms that underlie this accelerated loss of neuroplasticity in AD are still not completely understood. While the progressively increasing proteinopathy burden, such as amyloid β (Aβ) plaques and tau tangles, definitely contribute directly to a neuron’s functional demise, the role of non-neuronal cells in controlling neuroplasticity is slowly being recognized as another major factor. These non-neuronal cells include astrocytes, microglia, and oligodendrocytes, which through regulating brain homeostasis, structural stability, and trophic support, play a key role in maintaining normal functioning and resilience of the neuronal network. It is believed that chronic signaling from these cells affects the homeostatic network of neuronal and non-neuronal cells to an extent to destabilize this harmonious milieu in neurodegenerative diseases like AD. Here, we will examine the experimental evidence regarding the direct and indirect pathways through which astrocytes and microglia can alter brain plasticity in AD, specifically as they relate to the development and progression of tauopathy. In this review article, we describe the concepts of neuroplasticity and glial plasticity in healthy aging, delineate possible mechanisms underlying tau-induced plasticity dysfunction, and discuss current clinical trials as well as future disease-modifying approaches.

## Introduction

### Neuroplasticity Is Essential for Healthy Brain Function

In the late 1800s and early 1900s, the scientist Santiago Ramón y Cajal set down his Neuron doctrine which defined the neuron as the physiologic, metabolic, anatomic, and genetic basis of the nervous system (Ramón y Cajal, 1899/1904; Shepherd, 1991; Jones, 1994; Mateos-Aparicio and Rodríguez-Moreno, 2019). He also proposed that the brain can improve its function by improving the connections between neurons, establishing a paradigm that is now known as neuroplasticity (Ramón y Cajal, 1892, 1894a,b,c; Stahnisch and Nitsch, 2002). In 1949, Hebb (1949) formally defined neuroplasticity to refer to increased neuronal activity leading to stronger neuronal connections. Indeed, a simplified way to illustrate the Hebbian definition of neuroplasticity is through the statement: “cells that fire together, wire together” (Shatz, 1992). It follows that plasticity can be thought of as a continuum of neuronal activity that results in improved brain function through an iterative and cooperative process.

**Figure 1:**
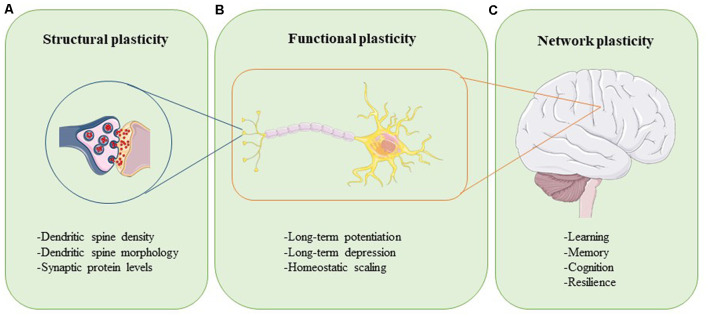
Neuroplasticity can be assessed at different levels. Structural plasticity refers to the morphological changes that occur at the synapse, such as altered dendritic spine density, dendritic spine shape, and synaptic protein profiles **(A)**. Functional plasticity affects neuronal circuit regulation and includes processes such as long-term potentiation (LTP), long-term depression (LTD), and homeostatic scaling **(B)**. At the organ level, network plasticity modulates learning, memory, cognition, and resilience **(C)**. Proper function and careful integration of all levels of plasticity are required for healthy brain function.

Plasticity can be assessed at different levels (Figure 1). Synaptic plasticity can be measured structurally at the level of altered dendritic spine morphology, which is modulated by synaptic activity and determines synapse strength (Figure 1A; Harris and Kater, 1994; Gipson and Olive, 2017). Experimental evidence in rats, mice, birds, squirrels, organotypic brain slices, and neuronal cultures have revealed that increases in neuronal activity lead to spine induction (Yuste and Bonhoeffer, 2001). Also, the size of a dendritic spine head correlates with the number of postsynaptic receptors and the number of presynaptic vesicles ready for release (Yuste and Bonhoeffer, 2001). The functional readouts of synaptic plasticity can be captured through long-term potentiation (LTP) and long-term depression (LTD), which are processes through which synaptic transmission is either strengthened or weakened, respectively (Figure 1B; Bear and Malenka, 1994). During LTP, synaptic activity is increased, unused dendritic spines are pruned, activated spines are stabilized and new spines are made (De Roo et al., 2008). The opposite happens in LTD: synaptic activity is reduced, synapses are weakened, and dendritic spines shrink or are lost completely (Gipson and Olive, 2017). Synaptic plasticity within a group of neurons is modulated through homeostatic scaling, a process in which neurons keep their output activity within a physiologic range to normalize overall signaling (Figure 1B; Chowdhury and Hell, 2018). Viewed at the level of large neural networks and the brain as an organ, plasticity affects phenomena such as cognition, learning, memory, and resilience (Figure 1C; Stampanoni Bassi et al., 2019). The brain is thus a very adaptable, self-regulating organ that is capable of rewiring itself, if necessary. However, the capacity of the brain to accomplish these tasks efficiently and effectively is impeded by aging and even more so in conditions like injury and neurodegenerative disease.

### Glia Are Integrally Involved in Neuroplasticity

The healthy functioning of neuronal networks in the brain requires non-neuronal cells as well (Allen and Lyons, 2018). Glial cells, such as astrocytes and microglia, are constantly interfacing with neurons to modulate and refine signaling besides providing trophic support (Allen and Lyons, 2018). The “tripartite” synapse model was established to describe the interactions between the presynaptic compartment, the post-synaptic compartment, and the perisynaptic astrocytic processes that surround them (Perea et al., 2009). As more information regarding the importance of microglia in neuronal signaling emerged, a “quadripartite” synapse model was proposed that includes perisynaptic processes from microglia along with the other components (Schafer et al., 2013). Due to their proximity to the synapse, glial cells are in a critical position to manipulate synaptic activity. Indeed, glial cells respond rapidly to changes in their environment in a matter of seconds (Nimmerjahn et al., 2005). Such stimulus-driven responses of these non-neuronal cells aim to preserve the homeostasis of the neuronal and synaptic milieu (Vainchtein and Molofsky, 2020). This quick response of the microglia and astrocytes to compensate or adjust their function can be thought of as glial plasticity. It can be hypothesized that the rapid alterations in glial plasticity may have a long-term and protracted effect on neuronal health and neuroplasticity. The mechanisms by which these non-neuronal cells regulate synaptic activity and plasticity are of immense interest because of the enormous impact these processes have on cognition and healthy brain function.

## Neuroplasticity Loss in AD and Related Tauopathies

**Figure 2:**
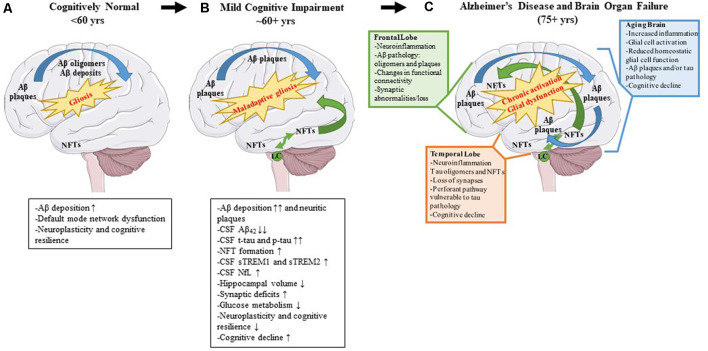
Pathological changes and biomarker alterations observed in the development of Alzheimer’s disease (AD). Moving left to right: schematic depicting brain alterations in a cognitively normal individual **(A)**, in an mild cognitive impairment (MCI) individual **(B)**, and in an end-stage AD patient **(C)**. Amyloid β (Aβ) pathology begins in the frontal cortex and spreads throughout the brain (blue arrows). Neurofibrillary tangle (NFT) pathology begins in the medial temporal cortex and locus coeruleus and spreads to the rest of the brain (green arrows). Gliosis increases with age, and the onset of the disease leads to maladaptive gliosis and eventual chronic glial activation and dysfunction. Biomarker alterations corresponding to each stage are listed under **(A,B)** in black boxes. Abnormal changes in the frontal and temporal lobes as well as changes observed in an aged brain are listed in **(C)** in green, orange, and blue boxes, respectively.

Alzheimer’s disease (AD) is a neurodegenerative disease characterized clinically by progressive memory loss, loss of executive function, confusion, disorientation, and aphasia (Alzheimer’s Association, 2020). Neuropathologically, AD is characterized by intracellular neurofibrillary tangles (NFTs) and extracellular amyloid β (Aβ) plaques (Braak and Braak, 1991). The pathophysiology of AD follows a stereotypical spatio-temporal pattern: extracellular Aβ plaque pathology appears first in the frontal lobes, even before the appearance of any clinical symptoms, followed by the appearance of NFT deposits in the transentorhinal cortex and hippocampus (Braak and Braak, 1991). Imaging studies investigating connectivity in these brain regions have shown that, even in preclinical or pre-pathological stages, subtle functional connectivity disturbances can be detected (Grieder et al., 2018; Tijms et al., 2018). For example, the default mode network (DMN) is affected even before robust Aβ plaque pathology sets in and presages the emergence of Aβ plaques (Palmqvist et al., 2017). This network includes hubs such as the medial prefrontal cortex, posterior cingulate cortex, retrosplenial cortex, basal forebrain, and thalamus (Alves et al., 2019). Aβ deposition leads to hypometabolism in brain regions connected by the DMN and this hypometabolism may ultimately lead to cognitive deterioration (Pascoal et al., 2019). It should also be mentioned that Aβ deposits are directly correlated with increasing microglial and astrocyte engagement (Selkoe and Hardy, 2016). It is possible, that since the healthy brain is fairly plastic, especially in younger ages, it can compensate for changes in neuronal connectivity, neuroinflammation, and initial Aβ proteinopathy, until a threshold is reached at which the compensatory alterations fail (Burke and Barnes, 2006; Figure 2A).

NFTs are complex intracellular deposits that appear decades after initial Aβ emergence. These intracellular inclusions are composed primarily of hyperphosphorylated forms of the protein tau which is a microtubule-binding protein that stabilizes microtubules (Iqbal et al., 2016). NFTs first appear in the entorhinal cortex and propagate to the hippocampus, the center of learning, and memory (Braak and Braak, 1991) initiating a toxic cascade of events leading ultimately to impaired cognition (DeKosky and Scheff, 1990). Several studies have shown that the severity of cognitive impairment correlates with intracellular NFT burden and neuritic plaque pathology and less with Aβ plaque pathology (Arriagada et al., 1992; Giannakopoulos et al., 2003; Figure 2B). During this time, the glial response is hypothesized to shift to a more toxic neurodegenerative phenotype characterized by a unique gene expression signature (reviewed in Deczkowska et al., 2018). This loss of glial plasticity, where these glial cells lose their protective function and assume a neurotoxic function, may be related to loss of neuroplasticity leading to neurodegeneration and cognitive failure in the end stage of the disease (Figure 2C). A key factor to consider is that many elderly people have Aβ deposits or even tau pathology in their brains and retain high levels of cognition (Price and Morris, 1999; Knopman et al., 2003; Crary et al., 2014). Because the brain is very adaptable, it can compensate for synaptic dysfunction or synaptic loss in the early phases by altering the properties of remaining synapses (Figure 2A). However, at some point, the ability of the brain to compensate is exceeded and efforts to stave off neuronal loss and cognitive decline are no longer viable (DeKosky and Scheff, 1990; Scheff et al., 1990; Figures 2B,C).

Since one of the hallmarks of AD is NFTs, AD is included under the umbrella of neurodegenerative diseases called tauopathies. In AD and other tauopathies, tau becomes hyperphosphorylated, dissociates from microtubules, and accumulates as intracellular inclusions of misfolded, non-functional protein. Such intracellular inclusions of aggregated tau protein are referred to as NFTs (Iqbal et al., 2016). The majority of tauopathies are sporadic, but familial frontotemporal dementia (FTD) has been linked to mutations in the gene that encodes tau (microtubule-associated protein tau, *MAPT*, Ghetti et al., 2015). FTD patients display severe frontal and temporal lobe atrophy and dementia (Ghetti et al., 2015). Tau-positron emission tomography (PET) tracing and ^18^F FDG (fluorodeoxyglucose)-PET studies show overlap between hypometabolism of glucose and tau deposition in the brains of FTD patients (Su et al., 2020). Also, iPSC neurons derived from FTD patients show abnormally increased activity when chronically depolarized (Sohn et al., 2019). While studies like these have begun to reveal altered synaptic function in FTD patients, pre-clinical models of tauopathy have provided us with mechanistic insight into how neuronal plasticity is affected by tau pathology. Reduction in spine density, a common feature of tauopathy mouse models, is associated with loss of function (Crimins et al., 2012; Sydow et al., 2016). It follows then that many mice expressing mutated forms of human tau display reduced levels of pre- and post-synaptic proteins (Schindowski et al., 2006; Yoshiyama et al., 2007; Crimins et al., 2012; de Calignon et al., 2012; Van der Jeugd et al., 2012). Interestingly, some of these changes are observed before the formation of NFTs (Yoshiyama et al., 2007). Some tauopathy models are characterized by electrophysiological abnormalities (Schindowski et al., 2006; Sydow et al., 2011; Crimins et al., 2012; Levenga et al., 2013; Polydoro et al., 2014) and others show non-convulsive epileptiform activity (Decker et al., 2016; Maeda et al., 2016). Mouse models deficient for tau have also provided information concerning synaptic plasticity. These studies complicate the picture since it seems that tau is involved in the breakdown of synaptic plasticity (as discussed above); however, it also seems to be necessary for maintaining these processes. A recent study found that *Mapt*^−/−^ mice display hyperactivity, altered LTP, and impaired memory, while a partial deletion of mouse tau (*Mapt*^+/−^) was able to partially rescue this phenotype (Biundo et al., 2018). Other studies have found similar results (Ikegami et al., 2000; Ahmed et al., 2014; Ma et al., 2014). Tau knockout mice also show both reduced LTD (Kimura et al., 2014; Regan et al., 2015) and impaired α-amino-3-hydroxy-5-methyl-4-isoxazolepropionic acid receptor (AMPAR) internalization (Regan et al., 2015). Finally, an underlying factor that is common across many tau transgenic models is increased neuroinflammation in the form of microgliosis and astrocytosis (Schindowski et al., 2006; Yoshiyama et al., 2007; de Calignon et al., 2012; Maeda et al., 2016). Indeed, a network-based analysis of neurodegenerative diseases based on prion models paired with cell-type classification showed that, during the development of the disease, the majority of the upregulated genes were expressed by microglia (Vincenti et al., 2015). These studies highlight the importance of understanding how tau-mediated changes to synaptic plasticity are intimately connected with glial plasticity alterations.

We do not fully understand how different factors, be they genetic or environmental, synergize, and predispose the brain to develop inexorable neurodegenerative diseases, such as tauopathies. Also, still unknown is how two seemingly disparate pathological proteinopathies, Aβ and tau, synergize and lead to loss of neuroplasticity observed in AD. A clear picture of how neuroplasticity and glial plasticity result in brain organ failure through a complex interaction between neurons, synapses, and non-neuronal cell function is only now emerging. Here we will review the known roles of non-neuronal cells—astrocytes, oligodendrocytes, and microglia—as they pertain to neuronal health and synaptic plasticity in AD and relate this to disrupted glial plasticity.

### Early Neuroplasticity Loss in AD and in Mouse Models Is Reversible

Synaptic dysregulation has been suggested to begin in the prodromal phase of AD, even before Aβ plaque formation (Walsh and Selkoe, 2007), perhaps triggered by oligomeric Aβ or transient soluble Aβ species that are neurotoxic (Heiss et al., 2017). Interestingly, not only does Aβ affect synaptic plasticity, other cleavage products of the amyloid precursor protein (APP), the parent molecule of Aβ, can also have neurotoxic functions. For example, the APP intracellular domain (AICD), produced as a by-product of the amyloidogenic processing of the APP protein, has been shown to regulate synaptic function and may also play a role in synaptic plasticity (Konietzko, 2012; Pousinha et al., 2017; Skaper et al., 2017). It is also noteworthy that soluble APPα (the most common proteolytic cleavage product of APP in healthy brains) has been suggested to play a role in synaptic growth and plasticity, but whether APPα is helpful or damaging remains highly debated (Corbett and Hooper, 2018).

The majority of studies seeking to improve synaptic plasticity have been conducted using either transgenic rodent models of Aβ deposition or injection models of Aβ (Hao et al., 2011; Bachstetter et al., 2012; Chen et al., 2012; Duffy and Hölscher, 2013; Wu et al., 2013; Deibel et al., 2016; Iaccarino et al., 2016; Ahmad et al., 2017; Wilkaniec et al., 2018; Liang et al., 2019; Martorell et al., 2019), while very few have been executed in tau transgenic models (Martorell et al., 2019), leading to a relative lack of understanding of tau-related abnormalities in neuroplasticity and glial plasticity. In wild type mice or human *APP* transgenic mice, various inhibitors, agonists, or small molecules as well as brain stimulation and environmental enrichment have been tested (Supplementary Table S1). In most cases, these manipulations resulted in improved performance in cognitive paradigms, increased synaptic function, increased the density of dendritic spines and increased levels of synaptic proteins (Hao et al., 2011; Bachstetter et al., 2012; Chen et al., 2012; Wu et al., 2013; Ahmad et al., 2017; Liang et al., 2019; Martorell et al., 2019). Many of these treatments reduced neuroinflammation (Hao et al., 2011; Bachstetter et al., 2012; Chen et al., 2012; Wu et al., 2013; Ahmad et al., 2017; Liang et al., 2019) and a few studies also reported improved neuronal survival (Chen et al., 2012; Ahmad et al., 2017). This suggests some form of synergy between processes connecting brain immunity, neuroplasticity, and neuronal survival. However, other studies involving cholinergic depletion and optogenetic stimulation did not result in improved neuroplasticity and sometimes even decreased cognitive performance and increased neuroinflammation (Deibel et al., 2016; Iaccarino et al., 2016). Effects on Aβ and tau neuropathology were mixed, with some studies showing reduced burden (Hao et al., 2011; Chen et al., 2012; Wu et al., 2013; Iaccarino et al., 2016; Ahmad et al., 2017; Martorell et al., 2019), while others reported no effect (Bachstetter et al., 2012; Deibel et al., 2016; Liang et al., 2019), and these changes did not always correspond to the studies that showed improved synaptic plasticity.

Synaptic plasticity has also been modulated in non-AD mouse models with various agents, neuropeptides, and exercise (Soto et al., 2015; Liu, Yi et al., 2018; Luo et al., 2019; Zhou et al., 2019). These studies showed improvements in synaptic protein levels, LTP, and cognitive performance as well as reduced neuroinflammation and, when assessed, neurodegeneration (Soto et al., 2015; Liu, Yi et al., 2018; Luo et al., 2019; Zhou et al., 2019). These studies are only some representative examples of findings in the field, but what can be observed is that much of the research on synaptic plasticity in AD has been conducted in rodent models that only accumulate Aβ plaque pathology (Pozueta et al., 2013). They do not take into account the effects of tau pathology on synaptic dysfunction and synapse loss, which are the major determinates of cognitive decline in AD.

### Chronic Glial Signaling Counteracts Glial Plasticity in Neurodegenerative Conditions

Neuroinflammation increases with age and is considered a normal part of brain aging (Cribbs et al., 2012). Aged brains are characterized by increased activation of glial cells and elevated levels of pro-inflammatory molecules and reduced anti-inflammatory mediators (Di Benedetto et al., 2017). Glial cells in an aged brain are primed for inflammatory responses and they are slow in returning to baseline following acute insults (Di Benedetto et al., 2017). Also, senescent glial cells have a reduced ability to perform their homeostatic functions (Di Benedetto et al., 2017), thus, increasing the vulnerability of the aging brain to proteinopathy-related injuries. Indeed, neurodegenerative disease is accompanied by progressively exacerbated neuroinflammation marked by astrogliosis and microgliosis (Serrano-Pozo et al., 2011; Di Benedetto et al., 2017). Topics of critical interest in the field are how does abnormal proteinopathy impact glial cell functionality, how does dysregulated glial function alter intraneuronal proteostasis, and finally, how do these alterations contribute to the progression of disease?

Glia can directly regulate short-term neuroplasticity in the synaptic cleft, either through homosynaptic connections or by modulating complex synaptic loops—thus they support neuronal activity at the cellular and network level (Perea et al., 2009; Schafer et al., 2013). Glia also possesses immense plasticity of their own as they can react within seconds to environmental threats to rebalance network homeostasis (Nimmerjahn et al., 2005). Since glial plasticity processes are critical in supporting and complementing neuronal health and function, glial plasticity and neuronal plasticity are integrated processes. In diseases such as AD, or even during aging, chronic gliosis can lead to loss of function (Hickman et al., 2013) whereby glia are unable to respond to challenges resulting in dyshomeostasis, morphological changes, and disrupted signaling (Bellamy et al., 2015). In the next sections of this review, we will discuss how tau accumulation in AD is related to loss of brain function.

### Experimental Models Show That NFT Pathology Correlates With Gliosis and Cognitive Decline

Relative to the effects of Aβ pathology, cognitive decline and neurodegenerative features are closely associated with NFT pathology in AD (Arriagada et al., 1992; Gómez-Isla et al., 1997; Giannakopoulos et al., 2003). Until recently, it was widely believed that NFTs deposited as intracellular inclusions are the toxic form of tau, but recent research has provided evidence that even soluble forms of phosphorylated tau may have a pathogenic effect on neuroplasticity (Fá et al., 2016; Ghag et al., 2018). The questions remain though, how do different forms of Aβ and tau affect neuroplasticity and glial plasticity, and what is the spatio-temporal relationship between these proteinopathies and dysfunctional plasticity? In this review, we will focus on tauopathy and its effects on neuroplasticity and glial plasticity.

**Figure 3:**
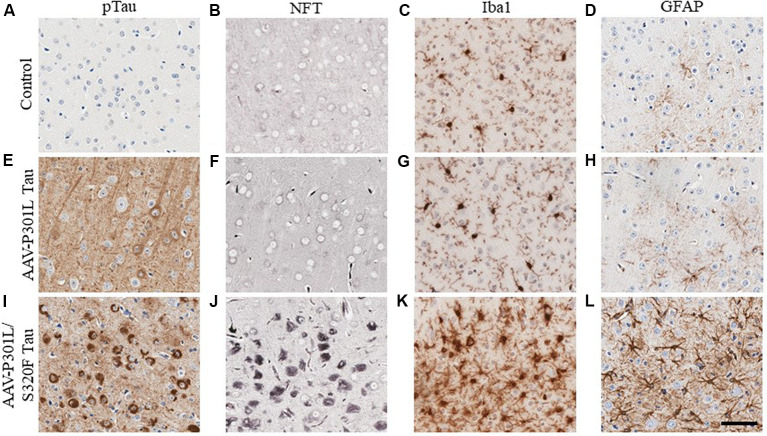
Phosphorylated tau vs. neurofibrillary tangle (NFT) tau species determine microglial and astroglial phenotype. Immunohistochemical detection of phosphorylated tau (CP13 antibody, pSer202 tau epitope), NFT tau (histological Gallyas silver stain), microglia (Iba1 antibody), and astrocytes (GFAP antibody) in the cortex of 3-month-old nontransgenic (C57/Bl6) mice that received neonatal intracerebroventricular delivery of adeno-associated virus (AAV) expressing P301L tau or P301L/S320F tau compared to age-matched naive C57/Bl6 control mice. AAV-P301L tau expressing mice accumulate intracellularly and neuropil phosphorylated tau (ptau; **E**), while in the AAV-P301L/S320F tau expressing mice, ptau is concentrated in the somatodendritic compartment **(I)**. No ptau is observed in control **(A)**. Histological Gallyas silver stain indicates frank NFT pathology only in the AAV-P301L/S320F tau mice **(J)**, and not in the AAV-P301L tau mice **(F)** or control group **(B)**. Detection of microglia (Iba1 antibody) and astrocytes (GFAP antibody) reveals increased hypertrophy of microglia and astrocytes in the AAV-P301L/S320F tau mice **(K,L)** compared to the AAV-P301L tau **(G,H)** and control mice **(C,D)**. Scale bar: 50 μm.

In AD, tau pathology originates mostly in the trans-entorhinal and hippocampal regions of the brain and then spreads out of the medial temporal lobe to other brain areas as the disease progresses (Mudher et al., 2017). This has been recapitulated in rodent transgenic models of tauopathy where the spread of tau pathology out of the medial temporal lobe involves the propagation of soluble tau species along synaptically connected neuronal networks (de Calignon et al., 2012; Calafate et al., 2015; DeVos et al., 2018). Another mechanism of tau spread involves the dispersal of tau pathology via glial cells (Asai et al., 2015; Hopp et al., 2018). Gliosis closely follows the development of tauopathy in transgenic mouse models (Ramsden et al., 2005; Schindowski et al., 2006; Yoshiyama et al., 2007; de Calignon et al., 2012; Maeda et al., 2016). We developed several mouse models of tauopathy in our lab to understand how gliosis is differentially regulated in the presence of intracellular phosphorylated tau and NFT inclusions (Koller et al., 2019). These models express different human mutant tau constructs following neonatal delivery of recombinant adeno-associated viruses (AAV). We observed a dramatic induction of gliosis, especially of hypertrophic microglia in rodents with NFT pathology but not in mice with somatodendritic hyperphosphorylated tau (Figure 3). Microglia present in NFT-rich regions display considerably hypertrophic morphology (Figure 3K) when compared to microglia in a mouse brain with soluble phosphorylated tau but no NFTs (Figure 3G) or microglia in control cohorts (Figure 3C). Astrocytes display similar changes (Figures 3D,H,L). This might reflect a continuum of glial functionality accompanying the spectrum of tau pathogenesis, starting from accumulation of relatively soluble hyperphosphorylated tau in the somatodendritic compartment towards more neurotoxic NFT deposits.

*In vivo* imaging studies of rTg4510 mice have shown that both pathological tau and neurodegeneration correlate with microglial activation (Sahara et al., 2018). Also, the elimination of senescent microglia and astrocytes can block the cognitive impairment and neurodegeneration observed in tauopathy mouse models (Bussian et al., 2018). However, glial cell responses are not always detrimental and are needed to protect the brain. In some instances, astrocytes can form protective scars (Sofroniew, 2009) and microglia can mount a rapid protective phagocytic response to neurotoxic proteinaceous species (Meyer-Luehmann et al., 2008; Sarlus and Heneka, 2017). These are reactive events designed to provide some resilience to the homeostasis of the neural network, but emerging evidence suggests that the glial response to progressive neuropathological disruption in neurons, as observed through morphological or functional changes, may be indicative of some sort of loss of glial plasticity when this reactive process leads to progressive neuro-glial toxicity rather than a compensatory healing outcome (Alibhai et al., 2018). Indeed, the presence of soluble forms of neurotoxic tau can trigger microglial senescence, reduce microglial surveillance, abort successful phagocytosis and clearance, and worsen neuro-glial homeostasis (Streit et al., 2009; Sanchez-Mejias et al., 2016).

Consistent with reports from other laboratories (Ramsden et al., 2005; Schindowski et al., 2006), we observed that mice with accelerated NFT deposits also developed rapid memory impairment (Koller et al., 2019). While our results were consistent with human data (reviewed in Nelson et al., 2012), other preclinical studies have shown that tau conformers, independent of NFT, underlie memory deficits and network alterations (Santacruz et al., 2005; Bolós et al., 2017; Green et al., 2019). The effects of accumulating proteinopathy on dendritic spine morphology and strength of synaptic connectivity may also be dependent on heterogeneous age-progressive spatial and temporally relevant factors, not just on the intracellular proteinopathy burden. How glial cell function and their evolving plasticity across the spectrum of age-progressive proteostasis contribute to such feedback or feedforward mechanisms to determine neuroplasticity and neurodegeneration remain of interest.

## Role of Non-Neuronal Cells in AD and Related Tauopathies

### Age and Neuroinflammation Increase the Vulnerability of the Brain to AD Pathologies

Following the development of Aβ deposits in the frontal cortices, tau pathology starts accumulating in areas distal to Aβ deposits, such as in the entorhinal cortex and hippocampus (Braak and Braak, 1991). The molecular determinants that trigger tau hyperphosphorylation and the development of NFTs in the entorhinal cortex and then the hippocampus are unknown. The amyloid cascade hypothesis posits that cellular changes downstream of the development of Aβ pathology initiate tau pathogenesis (Hardy and Higgins, 1992). However, since ptau has been identified in brainstem nuclei of individuals as young as 30 years of age, subtle changes in tau physiology may begin much earlier in life than had been previously thought (Braak and Tredici, 2011). The hippocampus is the site of early synaptic loss in AD (Scheff and Price, 2006; Scheff et al., 2006; Bastin et al., 2020) and it is this synaptic loss and the development of NFTs that correlate with the eventual cognitive decline (Terry et al., 1991; Arriagada et al., 1992; Giannakopoulos et al., 2003). The entorhinal cortex and the hippocampus are selectively vulnerable to aging-related changes, and the major synaptic pathway connecting these two brain regions, the perforant pathway, is especially vulnerable to tau pathology (García-Sierra et al., 2000, 2001; Thal et al., 2000). Additionally, the extent of tau pathology and synaptic loss in this pathway correlates with the degree of dementia, especially in the oldest old (García-Sierra et al., 2000; Thal et al., 2000; Robinson et al., 2014), consistent with the observation that aging is the major risk factor for AD type neurodegenerative changes.

**Figure 4:**
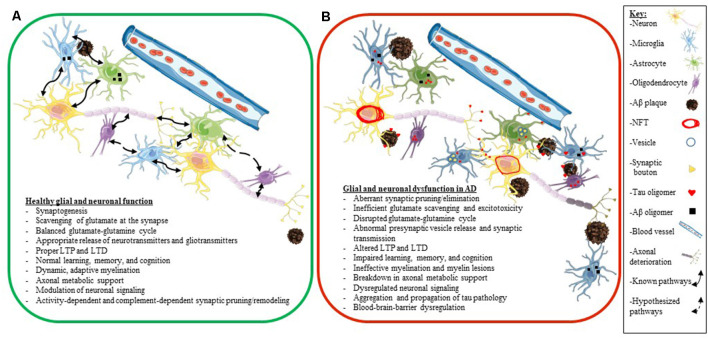
Glial and neuronal function and plasticity are dysregulated in AD. Schematics depicting healthy glial and neuronal function **(A)** compared to AD-induced glial and neuronal dysfunction **(B)**. Normal communication and support processes occur between neurons, astrocytes, microglia, and oligodendrocytes even in the presence of Aβ plaques and oligomers **(A)**. In AD, healthy communication and function break down between neurons, astrocytes, microglia, and oligodendrocytes **(B)**. Astrocytes and microglia take up tau, Aβ, and synaptic boutons; tau oligomers spread across synapses and travel between different cell types (neurons, astrocytes, microglia, and oligodendrocytes); neurons develop NFT deposits, and Aβ plaques convert to neuritic plaques and cause the death of neurites **(B)**. Listed below the schematics are dysregulated processes seen in a normally functioning brain **(A)** compared to an AD brain **(B)**. The key for the schematics is located on the right side of the figure.

A key factor to consider is that many elderly people have Aβ plaques in their brains and retain high levels of cognition (Price and Morris, 1999; Knopman et al., 2003). Also, while many people who accumulate tau pathology in their brain develop various tauopathies, other people have been shown to accumulate tau pathology during aging, with only a little or only mild cognitive decline (clinically diagnosed as primary age-related tauopathy or PART; Crary et al., 2014). Thus, we do not fully understand how different predisposing factors synergize to develop neurodegenerative diseases and how some people show greater resilience to proteinopathy and age successfully. Additionally, we also still do not fully understand how glial dysfunction and altered neuroplasticity and glial plasticity fit into the neurodegenerative cascade in AD. In the next few sections, we will explore the individual role of glial cells (astrocytes, microglia, and oligodendrocytes) as they pertain to neuronal health and synaptic plasticity in AD in more granular details (Figure 4).

### Astrocytes Are Not Just Supporting Cells in the CNS

Astrocytes are often described as the supporting cells of the nervous system. Emerging research has shown that they are not just the “glue” of the central nervous system (CNS), but that they are integral to many functional and physiological aspects of the nervous system (Allen and Lyons, 2018) including synaptogenesis (Chung et al., 2015). They facilitate the development of synapses through direct contact as well as through secreted factors such as thrombospondins, SPARCL1/Hevin, and cholesterol. They also regulate the localization of postsynaptic glutamate receptors through the release of glypicans (Allen and Eroglu, 2017). A new study showed that astrocytes are critical during the integration of adult-born hippocampal neurons into established neuronal circuits and the formation of dendritic spines on these neurons (Sultan et al., 2015). On the other hand, astrocytes also regulate synapse elimination (Allen and Eroglu, 2017). They can directly phagocytose synapses through Mertk and Megf10 receptors (Chung et al., 2013), or they can release cues such as SPARC and block synapse formation (Kucukdereli et al., 2011). They also can release TGFβ, which elicits increased C1q in neurons and marks synapses for phagocytosis by microglia (Schafer et al., 2012; Bialas and Stevens, 2013). Thus, astrocytes are dynamically related to synaptic homeostasis.

Astrocytes are not only involved in the structural maintenance of synapses; they also control their function as part of the tripartite synapse. Astrocytes regulate levels of glutamate at the synapse by taking up excess glutamate through their glutamate transporters (Stogsdill and Eroglu, 2017). Once they have removed glutamate from the synapse, astrocytes convert it to glutamine, which they provide to neurons to convert back to glutamate to be used for excitatory neurotransmission (the glutamate-glutamine cycle; Tani et al., 2014). This is a dynamic process, as high levels of neuronal activity are accompanied by increased glutamate transporters on astrocytic processes, while low activity levels result in a reduced number of glutamate transporters (Allen, 2014). Additionally, neuronal uptake of glutamate is also important for producing γ-aminobutyric acid (GABA) in GABAergic neurons and facilitating the function of inhibitory neurons (Schousboe et al., 2004). Therefore, astrocytes are indispensable in both excitatory and inhibitory signaling. Additionally, homeostatic synaptic scaling, a process for tuning neuronal network signaling, is regulated by TNFα secreted from glial cells (Stellwagen and Malenka, 2006).

Astrocytes directly participate in neuronal signaling by releasing gliotransmitters such as d-serine, glutamate, and adenosine triphosphate (ATP), each of which can affect neuronal activity and change synaptic transmission (Halassa and Haydon, 2010). In particular, d-serine is a co-agonist for N-methyl-D-aspartate receptors (NMDARs); therefore, the release of d-serine from astrocytes has the potential to modulate LTP (Henneberger et al., 2010). Providing further evidence for bi-directional communication between astrocytes and neurons, neurotransmitter release from neurons has been shown to cause local intracellular calcium increases in astrocytic processes (Di Castro et al., 2011) and astrocytes have been shown to regulate spike timing-dependent depression, a form of LTD (Min and Nevian, 2012), as well as neuronal excitatory postsynaptic currents (Pascual et al., 2012). Since LTP and LTD are important for learning and memory (Bliss and Collingridge, 1993; Abel and Lattal, 2001), this illustrates the critical role astrocytes in neuroplasticity. Indeed, astrocytic activation has been shown to increase hippocampal LTP, augment memory acquisition, and improve cognitive performance (Adamsky et al., 2018). Also, gene expression alterations in the hippocampus following learning indicate that there are changes in the energy metabolism of both astrocytes and neurons, further supporting the idea that memory formation requires concerted actions of neurons and glia (Tadi et al., 2015).

### Oligodendrocytes Provide Trophic Support to Axons and Modulate Neuronal Signaling

Oligodendrocytes are glial cells that wrap around neuronal axons and produce the myelin sheath, which acts as an insulator around the axon and facilitates rapid signal conduction. Although not the only contributing factor, it has been suggested that neuronal activity plays a role in myelination and that adaptive myelination is a form of plasticity since it allows for the fine-tuning of neuronal circuits and modulation of neuronal signaling (Foster et al., 2019). Aspects of myelination that can be modified include the thickness of the myelin, the size of the internodes, and the length of myelination between internodes (Waxman, 1980; Wu et al., 2012; Ford et al., 2015). Increased neuronal activity in the brain and spinal tracts has been shown to increase the differentiation of oligodendrocyte progenitor cells (OPCs) into mature myelinating oligodendrocytes and increased myelination affects neuronal circuitry and signal conduction in the CNS regions affected (Li et al., 2010; Gibson et al., 2014; Nagy et al., 2017; Mitew et al., 2018). Interestingly, it has been found that learning a new task enhances oligodendrogenesis in adult mice while inhibiting new oligodendrocyte production is detrimental to learning (Almeida and Lyons, 2017). Also, social isolation results in impaired myelination in mice resulting in deficits in sociability and working memory tasks (Almeida and Lyons, 2017). These studies highlight the importance of neuronal activity in myelination and demonstrate the necessity of dynamic myelination in learning and memory.

Another key aspect of the oligodendrocyte-neuron relationship involves metabolic coupling. Oligodendrocytes produce lactate and, using the MCT1 transporter, provide it to neuronal axons as an energy source (Fünfschilling et al., 2012; Lee et al., 2012). It has been suggested that this process is regulated by a neuronal activity because glutamate released by myelinated axons during action potentials activates NMDA receptors and increases Ca^2+^ in the surrounding myelin (Micu et al., 2016). Oligodendrocytic NMDA receptor activation leads to augmented trafficking of glucose transporter to the myelin sheath, increasing glucose uptake and allowing for the production of more lactate to support the energy needs of the axon (Krasnow and Attwell, 2016; Saab et al., 2016). This evidence provides further support that oligodendrocytes are instrumental in activity-dependent neuroplasticity.

### Microglia Are Dynamic Glial Cells That Regulate CNS Immunity and Directly Influence Neuroplasticity

Microglia are the innate immune cells of the brain. They constantly scan their microenvironment with their highly dynamic processes, phagocytose debris, and react to injuries or perceived threats (Nimmerjahn et al., 2005). In addition to their immune function, microglia play a critical role in processes that affect neuronal plasticity. For example, microglia have been shown to sense signals indicative of high neuronal firing rates and make contact with those active neurons to modulate their activity (Li et al., 2012). Also, ATP released by microglia causes astrocytes to release glutamate, activating neuronal metabotropic glutamate receptors and resulting in increased frequency of postsynaptic currents (Pascual et al., 2012). Microglia have even been shown to modulate neuronal circuit synchronization (Akiyoshi et al., 2018). Therefore, microglia surveil the brain not only to find debris and clear them away but also to correct abnormal signaling and prevent circuit failure (Akiyoshi et al., 2018). Based on the evidence that microglia interact at the level of synapses, they were added to the “tripartite synapse” model, modifying the model to include microglia as part of the “quadripartite synapse” (Schafer et al., 2013). As we have seen with astrocytes and oligodendrocytes, plasticity is a two-way street in microglia as well, as shown by multiple experimental models. Microglial motility is at least partially dependent upon signaling through their purinergic receptors activated by ATP/ADP (Koizumi et al., 2013).

A notable microglial receptor important for plasticity is the CX3C chemokine receptor 1 (CX3CR1). Hippocampal slices from *Cx3cr1* knockout mice display LTP deficits compared to hippocampi from wild type littermates (Rogers et al., 2011). Also, *Cx3cr1* knockout mice perform poorly in the Morris water maze and contextual fear conditioning paradigms, showing that signaling through CX3CR1 is important for plasticity and cognition (Rogers et al., 2011). Another study found that microglia support synapse formation during BDNF-dependent learning (Parkhurst et al., 2013). They showed that the post-synaptic density increased following learning of a motor task, and when microglia were ablated, new synapses were not established and mice did not learn the motor task (Parkhurst et al., 2013).

Microglia also directly affect the structure of synapses. During development, microglial synaptic pruning occurs to eliminate excess connections which are needed for healthy brain development (Paolicelli et al., 2011). Delayed synaptic pruning due to a reduction in microglia results in structural and electrophysiological abnormalities, which may play a role in developmental disorders (Paolicelli et al., 2011). It has been shown that synaptic pruning is mediated by the complement system (Fourgeaud and Boulanger, 2007; Stevens et al., 2007; Schafer et al., 2012; Stephan et al., 2012; Sierra et al., 2013) and is sensory- and activity-dependent (Tremblay et al., 2010; Schafer et al., 2012; Sierra et al., 2013). Also, a recent study found that triggering receptor expressed on myeloid cells 2 (TREM2) is necessary for synaptic pruning during development (Filipello et al., 2018), which is especially interesting since TREM2 variants have been linked to increased risk for AD and FTD (Guerreiro et al., 2013a,b; Filipello et al., 2018). We will discuss the function of TREM2 in more specific details in later sections.

In adulthood, synapses continue to undergo pruning (although to a lesser extent than during development) and remain dynamic, facilitating learning and memory as well as responding to injury (Zuo et al., 2005; Lohmann and Kessels, 2014). Interestingly, a recent study postulated that “synaptic pruning” may be too simple of a term for synapse removal as the researchers observed selective trogocytosis, or partial elimination, of the presynaptic compartment, but not the post-synaptic compartment in the hippocampus (Weinhard et al., 2018). They also observed the initiation of spine head filopodia at sites of microglia-synapse contact (Weinhard et al., 2018). Therefore, microglia have the capacity for extremely elegant synaptic remodeling and play a crucial role in regulating neuronal circuitry and plasticity (Weinhard et al., 2018). Another study has shown that intra-cortical injection of heat-killed bacteria leads to a process of “synaptic stripping” (Trapp et al., 2007), similar to that observed previously in the facial nucleus axotomy model (Blinzinger and Kreutzberg, 1968). This is a process mediated by microglia that removes synaptic connections of neurons by closely apposing the neuronal perikarya. Because there was no neurodegeneration, this process of synaptic elimination, where the microglia ensheathe the neurons, is considered a neuroprotective response. Microglia, when under the influence of glucocorticoid-induced stress, can also reduce spine density in a combinatorial mouse model of tau and Aβ (Pedrazzoli et al., 2019), suggesting a multi-system control of neuronal plasticity in the diseased brain. Additionally, in injury conditions, microglial function leads to a broader brain plasticity phenomenon, defined as “plasticity of plasticity” which is related to their response to modifying their morphology, function, and transcriptional identity in response to environmental threats (Banati, 2002). Such changes in microglia may induce neuroplasticity on a cellular and physiological scale. Overall, one of the major questions in the field exists around whether synaptic pruning, while usually a tightly regulated process, becomes dysregulated and if this aberrant pruning plays a role in neurodegenerative diseases (Stevens et al., 2007). Thus, more studies examining the spatio-temporal context of synaptic pruning by microglia are needed to improve our understanding of this process in both heathy and disease states.

## Glial Homeostasis and Plasticity Are Lost During Aging

### Aging Alters the Capacity of the Brain for Synaptic Plasticity

Age is the primary risk factor for neurodegenerative diseases such as AD (Alzheimer’s Association, 2020). The brain undergoes major changes just during the process of aging. Neurons of the aged brain display less complex dendritic arborization, shorter dendrites, and fewer dendritic spines (Dickstein et al., 2007). Also, cognitive domains such as processing speed, working memory, inhibition, and long-term memory tend to decline with age, while other processes such as knowledge storage and implicit memory are relatively preserved (Park et al., 2002; Park and Reuter-Lorenz, 2009). However, aging is a process that varies from person to person, with some people aging more “successfully” than others (Morrison and Baxter, 2012; Harada et al., 2013) and possessing higher levels of cognitive reserve even with an unfavorable proteinopathy burden (Harada et al., 2013). One method that our highly adaptive brains use to meet the challenges presented by aging is employing compensatory or alternative circuitry to overcome existing dysfunctional connections (Park and Reuter-Lorenz, 2009). This idea is supported by imaging studies that have shown over-activation and greater bilateral activation of the prefrontal cortex of aged adults (Park et al., 2001; Cabeza, 2002; Reuter-Lorenz, 2002). The brain does reach a threshold, however, at which the need for circuit reorganization exceeds its ability to adapt, at which point cognitive problems manifest (Burke and Barnes, 2006), especially since the aging brain is less efficient at compensating than the brain of a young person (Park and Reuter-Lorenz, 2009). Therefore, a large part of cognitive decline seen with aging can be attributed to the reduced ability of the brain to undergo synaptic plasticity (Morrison and Baxter, 2012). As age is the primary risk factor for neurodegenerative diseases like AD, much interest exists in exploring the effect of age on synaptic and glial plasticity in the brain.

### Aged Glial Cells Have Altered Inflammatory Profiles and Reduced Functionality

A recent transcriptomic study done in mice found that astrocytes take on a more reactive phenotype in normal aging and that this occurs in a region-specific manner in the brain: hippocampal and striatal astrocytes preferentially display large gene expression changes during aging (Clarke et al., 2018). Microglia also display age-dependent regional heterogeneity (Lawson et al., 1990; Grabert et al., 2016). A recent genome-wide analysis found that microglia in the young adult brain show a unique “immune-vigilant” phenotype in the hippocampus and the cerebellum, whereas, in the aged adult brain, these two brain regions diverge in their immunogenic phenotype: microglia in the cerebellum become increasingly more immune-vigilant while those in the hippocampus display the opposite effect (Grabert et al., 2016). Such age-dependent regional changes in microglial and astrocytic function may probably be an underlying factor in triggering regional vulnerability to lesions or injuries in a selective fashion observed in different neurodegenerative diseases.

Resting microglia in aged animals show a decreased capacity to survey their microenvironment and they display slower response to acute injury as well as a sustained, long-lasting response to chronic injury (Damani et al., 2011). Also, the “sensome,” or the collection of genes that encode the proteins used by microglia to surveille their surroundings, undergoes massive downregulation in aged mice compared to young adult mice (Hickman et al., 2013). Aged astrocytes are also deficient in their maintenance of brain homeostasis and function. Reactive astrocytes in an aged brain are deficient in supporting neuronal survival and neuronal function such as debris clearance or synaptogenesis (Liddelow et al., 2017). This reactive astrocytosis can be induced by the release of cytokines such as Il-1α, TNF, and C1q originating from microglia (Liddelow et al., 2017). Additionally this reactive astrocytic environment has been shown to not only be toxic to mature oligodendrocytes but also impair the differentiation of immature oligodendrocytes (Liddelow et al., 2017). These studies show that aged glial cells do not possess the capacity to perform their respective functions as effectively as those found in a young brain and this may make the brain more susceptible to injury or disease. These data also demonstrate the paramount importance of probing the neuron-astrocyte-microglia-oligodendrocyte nexus in aging and neurodegenerative disease to better understand the effect of neuroplasticity and glial plasticity on brain organ function.

## Glial Plasticity Is Altered in Neurodegenerative Disease

### Several Microglial Genes Increase the Risk of Developing AD and Other Tauopathies

Genome-wide association studies have identified risk variants for AD in several proteins expressed by microglia (such as TREM2, TREM1, APOE, PLCG2, ABI3, CR1, and CD33), further linking microglial function to AD and suggesting a role in disease pathogenesis (Sims et al., 2017; Hansen et al., 2018; Henstridge et al., 2019; McQuade and Blurton-Jones, 2019). Many of these genes are connected to Aβ metabolism, but how they affect tauopathy remains unanswered. Specifically, here we will discuss the role of TREM2 and TREM1 as well as their relationship to Aβ, tau, and cognitive decline in neurodegenerative proteinopathies.

TREM2 variants have been associated with increased risk of developing AD (Sims et al., 2017; Carmona et al., 2018) as well as FTD and other neurodegenerative diseases (Guerreiro et al., 2013a,b; Yeh et al., 2017). For example, AD patients with the R47H mutation in TREM2 display earlier age of onset and shorter disease duration (Slattery et al., 2014; Korvatska et al., 2015). Also, (R47H) TREM2 patient brains display fewer microglia around Aβ plaques, indicating altered microglial function and increased axonal dystrophy (Yuan et al., 2016). In the cerebrospinal fluid (CSF) of AD patients, soluble TREM2 (sTREM2) levels are elevated when compared to healthy controls (Brosseron et al., 2018) and increase with disease progression (Suárez-Calvet et al., 2016a). Although this elevation is not reliable enough for AD diagnosis, it may be indicative of microglial activity (Suárez-Calvet et al., 2016b; Brosseron et al., 2018). New data suggests that increased CSF sTREM2 is correlated with tau-related neurodegeneration, while decreased CSF levels of sTREM2 are associated with Aβ pathology (Suárez-Calvet et al., 2019). While the (R47H) TREM2 mutation is not linked with FTD, biallelic mutations in TREM2 have been associated with FTD (Guerreiro et al., 2013a; Slattery et al., 2014). These findings warrant more research into the association between TREM2 function, neuroplasticity, and tau abnormalities.

Pre-clinical studies exploring the effect of TREM2 in tauopathy have not been entirely conclusive. One group showed that tau transgenic mice lacking *Trem2* display reduced inflammation and neurodegeneration with no alterations in tau phosphorylation or solubility (Leyns et al., 2017), while another group observed that tau phosphorylation was increased in tau transgenic mice deficient for *Trem2* (Bemiller et al., 2017). Of note, these studies were conducted in different tauopathy mouse models and the mice were of different ages, which may suggest an age-dependent effect of Trem2, which has been observed when assessing the effect of Trem2 deficiency on Aβ pathology (Jay et al., 2017). Additionally, in a TREM2 overexpression model, tau mice showed attenuated tau hyperphosphorylation, neuronal death, and synaptic loss as well as improved spatial cognitive performance (Jiang et al., 2016a). The authors attributed these results to the reduced neuroinflammation that they observed in these mice (Jiang et al., 2016a). How TREM2-mediated glial activity is mechanistically related to neuroplasticity remains entirely unexplored.

Recently, TREM1 variants have also been associated with AD. This variant is associated with increased Aβ burden as well as exacerbated cognitive decline in humans with AD (Replogle et al., 2015; Liu et al., 2020). A study of Aβ-PET imaging in cognitively normal individuals, MCI individuals, and AD patients that was conducted in the ADNI cohort showed that the TREM1 variant investigated was associated with MCI and was even more strongly linked with AD (Liu et al., 2020). Interestingly, assessment of the plasma of AD patients led to the finding that soluble TREM1 (sTREM1) levels were elevated, and concentrations of sTREM1 correlated to disease progression in AD patients (Jiang et al., 2019). Additionally, plasma sTREM1 levels also correlated positively with the amount of total tau found in AD plasma and this relationship was stronger in severe AD cases (Jiang et al., 2019).

Pre-clinical studies of TREM1 have mainly focused on Aβ pathology. An *in vitro* study found increased Aβ burden was likely due to impaired Aβ phagocytosis (Jiang et al., 2016b). These findings were supported by evidence showing that TREM1 deficiency in *APP*/*PSEN1* mice leads to increased Aβ42 levels and overall Aβ burden (Jiang et al., 2016b). Also, TREM1 replacement by microglial overexpression as well as activation of TREM1 signaling reversed Aβ pathology and ameliorated cognitive deficits (Jiang et al., 2016b). The relationship between TREM1 and tau pathology has yet to be explored in-depth, although, one study found that TREM1 is elevated in cortical lysates from the h-tau tauopathy mouse model (Garwood et al., 2010). In the light of these results, the importance of TREM2 and TREM1 in AD therapies is being increasingly recognized as timely, spurring research into these targets (Saadipour, 2017; Carmona et al., 2018).

### Microglia Phagocytose Disease-Associated Synapses and Propagate Tau Pathology

Tau associates with presynaptic vesicles via its amino-terminal domain and hinders vesicle release (Zhou et al., 2017). Moreover, the complement protein C1q is found extracellularly adjacent to the post-synaptic density in older tau transgenic mice (Dejanovic et al., 2018) and helps microglia engulf synaptic material in a C1q-dependent manner (Dejanovic et al., 2018). Treatment with an anti-C1q antibody inhibited microglial engulfment of synaptic material, prevented the loss of synapses, and also boosted synaptic density (Dejanovic et al., 2018). This study highlights the idea that tau-induced microglial phagocytosis of synapses leads to synaptic loss (Dejanovic et al., 2018). Another study showed that complement-dependent microglial phagocytosis can destroy synapses between engram cells, thus destroying encoded remote memories (Wang et al., 2020). These newly emerging studies form the foundation of our recent understanding of how microglial function is related to synaptic function and learning and memory.

Phagocytosis of tau-laden synapses may also have a downside, as additional evidence has shown that microglia are involved in the propagation of tau. We know that tau can spread from neuron to neuron along with synaptic connections (de Calignon et al., 2012; Calafate et al., 2015; DeVos et al., 2018) and is released in synaptic vesicles (Pooler et al., 2013). Tau can also be transmitted through exosomes (Saman et al., 2012; Polanco et al., 2016), ectosomes (Dujardin et al., 2014), or tunneling nanotubes (Abounit et al., 2016; Tardivel et al., 2016). It has also been shown that microglia phagocytize tau and secrete it in exosomes that then can be taken up by neurons, thereby spreading tau to neurons that may not be synaptically connected (Asai et al., 2015). Another study found that tau contained in microglia does indeed have “seeding” ability and can induce tau accumulation in recipient cells (Hopp et al., 2018). Further supporting the importance of microglia in tau transmission, microglial ablation following tau expression in the entorhinal cortex resulted in reduced tau phosphorylation and propagation to the dentate gyrus (Asai et al., 2015). This effect may be based on the amount of microglia that are removed from the brain, as partial microglial depletion (~30%) did not alter tauopathy in rTg4510 mice (Bennett et al., 2018a). A better understanding of whether microglial phagocytosis of tau-laden synapses contributes to the propagation of tau pathology could lead to the discovery of new modes for targeting tauopathy at the level of synapses.

### Astrocytes Accumulate Tau Pathology in Aging and Neurodegenerative Disease

Astrocytes also undergo morphological and functional changes in response to tauopathy. Indeed, 50% of humans aged 75 years and older show tau accumulated in the cell bodies and processes of astrocytes (Schultz et al., 2004). Glial tau pathology is not usually associated with AD, therefore, it has been suggested that this effect is likely due to aging (such as that found in aging-related tau astrogliopathy or ARTAG), although other tauopathies are characterized by glial tau pathology (Schultz et al., 2004; Lace et al., 2012; Kovacs et al., 2016). Types of inclusions in other tauopathies include astrocytic plaques in corticobasal degeneration (CBD), tufted astrocytes in progressive supranuclear palsy (PSP), and globular astrocytic inclusions in globular glial tauopathy (Kovacs, 2015). Even though the human brain expresses six different isoforms of tau (Sergeant et al., 1997), certain isoforms accumulate selectively in astrocytes compared to neurons in these different tauopathies (Ferrer et al., 2014). Also, tufted astrocytes in PSP and astrocytic plaques in CBD are labeled by antibodies specific to the carboxyl-terminus and the middle portion of the tau protein but are not detected by antibodies against the amino-terminus, suggesting that a portion of the amino-terminus of the tau protein may be missing in astrocytic tau pathology (Ferrer, 2018). On the other hand, in the thorn-shaped astrocytes found in ARTAG, tau is labeled at the carboxy-terminus, the middle portion of tau, and the amino-terminus (Ferrer et al., 2018). This raises questions about whether different types of astrocytic tau pathology are composed of different isoforms or truncations of tau protein.

Another unknown is whether the tau found in astrocytic inclusions originates from an external source, such as neurons, or if astrocytes express tau under some circumstances (reviewed in Kovacs, 2020). However, as only a couple studies have found evidence of astrocytes expressing tau (Shin et al., 1991; Miyazono et al., 1993), it is fairly widely accepted that astrocytes do not express tau, or if they do, it is expressed at very low levels. We have already discussed that astrocytes can phagocytose synapses (Kucukdereli et al., 2011; Chung et al., 2013; Allen and Eroglu, 2017) and we know that tau can be released from neurons (Pooler et al., 2013). Therefore, the proximity of astrocytes to synapses allows them to uptake extracellular tau released from neurons as shown by some groups (Perea et al., 2019). Also, astrocytic tau is seed-competent, as sarkosyl-insoluble brain homogenate from ARTAG brains was shown to seed tau pathology in neurons, astrocytes, and oligodendrocytes in the hippocampus of wild type mice (Ferrer et al., 2018). Therefore, astrocytes, as well as microglia, can spread tau in the brain. Additionally, while AD brain homogenate only seeded intraneuronal tau pathology, CBD and PSP brain homogenate led to oligodendrocytic, astrocytic and neuronal tau pathology, supporting the idea that different strains of the tau protein found in different tauopathies have unique cell-type specific pathogenic properties (Narasimhan et al., 2017). This is supported by another study that reported similar results, showing that injection of brain extracts from several different tauopathies (AD, tangle-only dementia, PSP, CBD, PiD, and argyrophilic grain disease) into ALZ17 mice or non-transgenic mice led to the formation of silver-positive tau inclusions and that the type of tau inclusion formed differed depending upon the type of brain extract injected (Clavaguera et al., 2013). Finally, another study showed an injection of CBD or AD brain extracts in young PS19 mice led to the development of tau pathology specifically in oligodendrocytes and neurons, respectively, and this cell-type specificity was noticed even in brain regions distal from the site of injection indicating that the biological nature of the original seeds was preserved during transmission (Boluda et al., 2015). Interestingly, the astrocytic inclusions found in PSP and CBD have been preferentially observed near blood vessels (Shibuya et al., 2011). In a study conducted in the GFAP-tau transgenic model that accumulates astrocytic tau, tau was observed in astrocytic end-feet abutting the blood-brain-barrier (Forman et al., 2005). As neurodegenerative diseases have been associated with a blood-brain-barrier breakdown (Zhao et al., 2015), this is a decidedly interesting observation with wide-reaching implications regarding the impact of tauopathy on the integrity of the blood-brain barrier (Bennett et al., 2018b).

### Breakdown of Astrocytic Support and Synaptic Modulation in Tauopathy

In AD, brain glucose uptake is impaired as shown by PET studies (Bischof et al., 2016). A recent study found that astrocytes comprise a significant part of the *in vivo* ^18^F-FDG PET signal (Carter et al., 2019). In a longitudinal study of familial AD patients, they showed that decreased astrocyte function, as assessed by ^11^C-deuterium-L-deprenyl (^11^C-DED) binding, aligned with reduced ^18^F-FDG PET uptake representative of glucose metabolism (Carter et al., 2019). Also, in a study of post-mortem brains, it was found that cognitively normal individuals with AD pathology exhibited enhanced expression of GLT-1 compared to cognitively impaired individuals with AD pathology, suggesting a protective role of glutamate transport in the brain (Kobayashi et al., 2018). Other studies also showed that AD patient brains display reduced glutamate transporters (GLAST and GLT-1) and vesicular glutamate transporter (VGLUT1; Masliah et al., 1996; Jacob et al., 2007). This reduction in glutamate transporters was associated with increased excitotoxicity and neurodegeneration, which suggests that deficient clearance of glutamate from the synapse leads to excessive activation of glutamate receptors (Masliah et al., 1996). AD brains also display lowered levels of enzymes involved in the metabolism of glutamate and GABA as well as those involved in the inter-conversion of glutamate to glutamine (Burbaeva et al., 2014). Also, the PS19 tauopathy model, which expresses FTD-associated P301S mutant tau, shows abnormal levels of glutamate and glutamine in the parahippocampal and hippocampal regions of the brain, respectively, in an age-dependent manner (Vemula et al., 2019). In a mouse model of astrocytic tauopathy (the GFAP-tau model), mice exhibited impaired motor function along with the accumulation of astrocytic tau and reduced expression of GLT-1, which aligns with what is observed in human CBD brains (Dabir et al., 2006). These studies show the dramatic dysregulation of the glutamate-glutamine cycle, functionally linking neurons and astrocytes in AD and other tauopathies. It may follow then that neuronal function and plasticity are directly modified by loss of astrocytic plasticity. *In vitro* experiments demonstrated that astrocytes that internalize tau oligomers display altered spontaneous intracellular Ca^2+^ transients and reduced release of gliotransmitters, including ATP (Piacentini et al., 2017). This causes lowered presynaptic vesicle release and depressed synaptic transmission in adjacent neurons (Piacentini et al., 2017). Altered synaptic transmission has implications for learning, memory, and cognition. Indeed, in AD, although LTD is relatively stable, LTP is impaired (Koch et al., 2012). Therefore, irregular glutamate-glutamine metabolism and transport paired with abnormal levels of neurotransmitters result in altered neuronal health and signaling that adversely impacts synaptic plasticity.

### Oligodendrocytes Accumulate Tau Pathology and Display Tau-Related Dysfunction

Some tauopathies are characterized by oligodendrocytic tau pathology in the form of coiled bodies or threads (Ferrer et al., 2014). Interestingly, Braak observed that the pattern for myelination during development inversely correlates to the pattern of NFT development and spread in AD (Braak and Braak, 1996). This observation has been corroborated by more recent findings indicating that white matter degeneration in AD follows the pattern of myelogenesis in reverse (Stricker et al., 2009). These studies suggest that those neurons that were last myelinated may be more susceptible to pathological changes in AD (Benitez et al., 2014).

Additionally, tau is transported in the white matter of the brain as well as the gray matter (Ferrer et al., 2019). Unilateral injections of brain fractions isolated from several different human tauopathy brains into the corpus callosum resulted in coiled body and thread tau pathology in both the ipsilateral and contralateral corpus callosum, indicating seeding and spread of tau pathology in oligodendrocytes (Ferrer et al., 2019). Also, increased levels of hyperphosphorylated tau in the cortex of AD brains predicted the severity of white matter hyperintensities seen in magnetic resonance imaging (MRI), which denote lesions usually caused by demyelination and/or axonal loss (McAleese et al., 2015). White matter hyperintensities have been shown to correlate with cognitive decline in patients with MCI (Tosto et al., 2014) as well as in those with dementia in general (Brickman et al., 2014). Since we know that myelination is important for neuronal plasticity and learning, these white matter lesions have great potential to disrupt neuronal signaling and negatively influence synaptic plasticity.

Also, inflammatory conditions, such as those seen in an AD brain, are sufficient to impair remyelination and stop OPCs from differentiating into mature oligodendrocytes (Miron et al., 2013). It is not surprising then that several genetic risk factors for AD are expressed in oligodendrocytes or OPCs such as *CLU*, *BIN*1, *ZCWPW*1, and *EPHA*1 (Henstridge et al., 2019). Also, oligodendrocytes are especially vulnerable to glutamate excitotoxicity, which is a feature of lost astrocytic homeostasis (Matute et al., 2007). Also, it has been shown that regenerated oligodendrocytes require astrocytes to form new myelin (Liu, Yiting et al., 2018). These studies strongly argue for a multi-system loss of plasticity in AD-type neurodegenerative diseases. These findings once more emphasize the importance of the elegant integration of different cell types in the CNS and illustrate how easily the loss of homeostasis in one cell type can drastically harm the other cell types, which in turn, has consequences for brain organ homeostasis.

## Therapeutic Approaches to Targeting Synaptic Plasticity

Throughout this review, we have discussed glial contributions to neuroplasticity and the elegant interwoven functions of astrocytes, microglia, and oligodendrocytes in the healthy brain. We have also discussed how these systems are altered by age and by tau proteinopathy, leading to a loss of overall plasticity. Currently, multiple clinical trials are underway to assess new therapeutic approaches targeting tau, though there are no disease-modifying therapies or prophylactics for tauopathies yet. Some treatments, such as monoclonal tau antibodies and active tau vaccines are showing early promise (Cummings et al., 2019; Jadhav et al., 2019), but an effective tau treatment or a cure for AD and related dementias remain elusive.

All the treatments currently used in the clinic for AD are neuroplasticity modulators while few address the underlying proteinopathy (Weller and Budson, 2018). It is possible that since glial homeostasis and synaptic plasticity are intimately connected, new therapeutics should target glial plasticity to combat proteinopathy and improve synaptic plasticity, cognition, and quality of life. Interestingly, as described in the next sections, some neuroplasticity modulators also affect glial function, which highlights potential cooperative interactions between neuronal and glial plasticity. Described below are a selection of some of the current and past clinical trials targeting synaptic plasticity and cognition.

### Brain Stimulation and Cognitive Training Improve Cognitive Function

Several types of brain stimulation including transcranial Direct Current Stimulation (tDCS) and Transcranial Magnetic Stimulation (TMS) have been shown to improve cognition in MCI and AD patients (Supplementary Table S2). tDCS has been associated with modulation of cortical excitability and synaptic connections and has been suggested as a potential therapy in multiple diseases and disorders because it is non-invasive and painless and has minimal or mild side effects (Stagg and Nitsche, 2011; Elder and Taylor, 2014). tDCS was shown to improve cognitive performance in healthy elderly individuals and reduce hyperexcitability in the stimulated brain regions (Meinzer et al., 2013). Although a single session of tDCS does not cause long-lasting alterations, it has been found that repeated tDCS sessions result in progressive and long-lasting improvement (Reis et al., 2009; Stagg and Nitsche, 2011). Also, fMRI assessment of the brains of these older individuals undergoing treatment showed that the treatment reversed the connectivity pattern to that matching younger brain performance characteristics (Meinzer et al., 2013). Acute assessment of tDCS in MCI patients revealed that MCI individuals were able to reach a similar performance level as healthy controls in a semantic word-retrieval task, a task in which performance worsens early on in dementia, accompanied by restoration of resting-state connectivity (Meinzer et al., 2015). As AD is associated with the altered function of several brain networks (DMN, control network, dorsal attention network, sensor-motor network, and salience network, among others; Brier et al., 2012), tDCS could be a potential mechanism to improve cognitive functioning in AD and MCI on a global scale (Flöel, 2014).

Another method of brain stimulation is TMS. TMS can be conducted either with a single pulse, paired-pulse, or repetitive stimulation (Elder and Taylor, 2014). The most common side effect of TMS is transient pain, but TMS is non-invasive and has been assessed to be safe (Elder and Taylor, 2014). TMS has been used in older adults to improve different types of memory and to ameliorate neuropsychiatric conditions such as depression (Elder and Taylor, 2014). There are several clinical trials underway to assess the efficacy of different types of TMS in AD, and repetitive TMS has been shown to result in improved performance during a naming task in AD patients (Cotelli et al., 2008; Supplementary Table S2). Brain stimulation is an effective intervention for improving cognition as it has minimal side effects and does not involve an invasive procedure. Additionally, clinical trials are attempting to enhance the cognitive outcomes of brain stimulation by pairing it with various cognitive training tasks.

Cognitive training can include computer-based training, the learning of particular tasks, or musical training, among others, and these interventions have already shown promise in MCI and AD (Supplementary Table S2). For example, a study in MCI patients found that a 12-week computer-based training program targeting memory and attention resulted in improved performance on recognition and recall tasks and found that this improvement in functioning lasted for 6 months post-training (Herrera et al., 2012). Another study assessing hippocampal activation as well as verbal memory in MCI patients showed that cognitive training involving auditory-verbal perception improved the patients’ ability to remember and their overall hippocampal function (Rosen et al., 2011). To improve experience-induced neuronal plasticity, older adults at risk for developing AD participated in 13 weeks of cognitive training targeting attention and working memory (Leung et al., 2015). These two cognitive domains were improved following the training period, while other cognitive domains, such as verbal or visual-spatial memory were not affected, suggesting experience-dependent plasticity (Leung et al., 2015). In a study assessing mild and moderate AD patients, individuals were trained on a structure-from-motion task. Both groups were impaired on the task, but the mild AD group was able to improve their performance after several training sessions, while the moderate AD group was unable to improve (Kim and Park, 2010). These results suggest that several cognitive domains that decline in MCI and AD remain plastic and can be re-trained; however, these studies also show that some domains can change only during the early stages of the disease, after which they cannot be rescued (Kim and Park, 2010).

Several clinical trials are assessing whether brain stimulation alone or in combination with cognitive training could be used to treat MCI and AD (Supplementary Table S2). For example, in one ongoing study, older individuals with mild neurocognitive disorder due to AD will be subjected to tDCS with working memory training or with tDCS alone (Cheng et al., 2015). The hypothesis is that the modulatory effect of tDCS will have an enhancing effect on working memory performance in the adaptive N-back task (Cheng et al., 2015). As this study and the other clinical trials combining brain stimulation and cognitive training are ongoing, the results remain to be determined.

### Physical Activity and Cognitive Training Prevent Cognitive Decline and Improve Cognition

Physical fitness training, aquatic exercise, “exergames,” as well as dancing have been suggested as potential therapeutics for AD. In a 6 month study, a resistance training exercise in women with probable MCI led to improved attention, memory, and brain plasticity (Nagamatsu et al., 2012). In a different study with both aquatic- and land-based exercise that was conducted for 16 weeks in elderly women, plasma Aβ and HSP27 levels were increased and vascular health was improved in these women (Kim et al., 2018). As cognitive training has also been shown to improve cognitive functioning, some studies have combined physical activity and cognitive exercises to enhance functional outcomes. For example, a trial using “exergames” (virtual reality–enhanced exercise) in MCI patients posited that since exercise can lead to cognitive improvement, a regimen of stationary cycling paired with virtual reality tours (cyber cycling) would increase participation and enhance cognition (Anderson-Hanley et al., 2012). The results of this study revealed that cyber cycling improves executive functioning and reduces the risk of MCI progression (Anderson-Hanley et al., 2012). Another study that combined physical training and cognitive training over 8 weeks found that this training reduced delta, theta, and beta rhythms, induced functional plasticity, and improved cognitive scores (Styliadis et al., 2015). Therefore, combinatorial therapies pairing exercise with cognitive training show promise for modifying cognitive decline in patients at risk for dementia.

### Pharmacological Interventions Leading to Improved Cognitive Function

One method for drug discovery is to test treatments that have already been approved for use in other diseases. A glucagon-like peptide 1 (GLP-1) analog called Liraglutide has been used to treat obesity and diabetes and is being assessed in an ongoing clinical trial to improve brain glucose metabolism as well as cognition in patients with mild AD (Femminella et al., 2019). A previous clinical trial had evaluated the efficacy of daily administration of Liraglutide in AD patients over 6 months and found improved glucose metabolism, but no change in cognitive scores or amyloid load (Gejl et al., 2016). The researchers suggested further study of this compound and cited the small number of participants and the short treatment window (Gejl et al., 2016). An ongoing Liraglutide trial has incorporated an extended treatment window of 12 months and recruited a substantially larger cohort of study participants (Femminella et al., 2019).

Another clinical trial assessed the effectiveness of the dopamine D_2_ agonist, rotigotine (RTG), in improving synaptic plasticity in patients with mild AD by altering cortical excitability and cholinergic transmission (Martorana et al., 2013). A paired-pulse paradigm was used to investigate the effect of RTG on cortical excitability by assessing intracortical inhibition, and facilitation and short-latency afferent inhibition was used to evaluate the effect of RTG on cholinergic transmission. Cortical excitability increased and cholinergic transmission returned to the baseline seen in the control subjects (Martorana et al., 2013). A similar RTG study evaluated the effect of RTG on cortical plasticity using transcranial stimulation with theta-burst stimulation (TBS) to induce LTP or LTD in mild AD patients (Koch et al., 2014). Cortical plasticity was improved and an additional assessment with short-latency afferent inhibition revealed increased cholinergic activity as well (Koch et al., 2014).

A study targeting the cholinergic system using Donepezil, an acetylcholinesterase (AChE) inhibitor, along with cognitive training has been done in AD patients (Gonzalez Rothi et al., 2009). AChE inhibitors increase the amount of acetylcholine at the synapse and improve cholinergic signaling. This tends to slow the decline of cognition in AD, but not necessarily reverse the cognitive impairment (Gonzalez Rothi et al., 2009). In this study, the premise was that if cognitive training was paired with increased cholinergic signaling, then cognition might improve and not just remain stable (Gonzalez Rothi et al., 2009). Assessment of performance revealed that half of the participants showed improvement on the verbal naming task, showing that this combined treatment is only partially effective (Gonzalez Rothi et al., 2009).

### Multimodal Therapies or Changes in Lifestyle Could Improve Overall Brain and Bodily Function

A few studies have combined more than two modes of treating cognition and synaptic plasticity and have implemented a lifestyle change regimen. A study involving a dietary regimen, physical activity, cognitive intervention, and a specific dietary supplement (epigallocatechin gallate) is underway to assess changes in function and structural connectivity in the brain. Another study has found that aerobic and resistance training, cognitive training, and vitamin D supplement have potential as a synergistic intervention to enhance mobility as well as cognition in older adults, especially those with MCI (Montero-Odasso et al., 2018). If these trials do establish the efficacy of a combined approach for slowing the loss of cognitive function or even reversing cognitive decline, then this would be an exciting step in the direction of using multimodal treatments to overcome the challenges presented by age-related dementia, MCI, and AD.

### Neuroplasticity Targeted Therapies Resolve Gliosis in Mouse Models

If neuronal and glial plasticity are connected, possibly these neuroplasticity targeted therapies could also ameliorate glial dysfunction or at least stabilize their function to support overall maintenance of brain organ homeostasis. Such a scenario would have a translational impact, when combined with promising therapies targeting the underlying proteinopathies in AD and related tauopathies (https://www.alzforum.org/therapeutics/aducanumab and https://www.alzforum.org/therapeutics/aci-35; retrieved June 8, 2020). In mouse models of AD-type amyloidosis, most of the environmental enrichment strategies, such as physical exercise and brain stimulation, have resulted in reduced Aβ and neuroinflammation (Iaccarino et al., 2016; Xu et al., 2016; Adaikkan et al., 2019; Martorell et al., 2019). Even in models that do not deposit Aβ, manipulating microglia could rescue effective behavior and induce adult neurogenesis following environmental enrichment (Ortega-Martinez et al., 2019). Interestingly, in wild type mice, such interventions have resulted in sustained effects on glial activation by recruiting microglia and upregulating NF-KB/MAPK pathways (Garza et al., 2020), suggesting that these manipulations can alter glial plasticity independent of their effects on AD-type proteinopathy. Due to the paucity of research on tauopathy using these manipulations, the relationship between these therapeutics and tau-mediated alterations in neuronal and glial plasticity remains unknown. Pharmacological interventions such as GLP-1 can also restore neurite complexity, dendritic spine morphogenesis, and spine development in non-AD experimental models (Yoon et al., 2020). Its effect on brain plasticity in Aβ models mostly show positive regulation (Cai et al., 2014; McClean et al., 2015), though the effect on tauopathy-related brain dysfunction remains unclear.

## Conclusion

Here, we have broadly described the impact of glial dysregulation on synaptic plasticity in AD and highlight the concept of “plasticity of plasticity” in the context of neurodegenerative dementias such as AD and related tauopathies. Experimental models have shown that synaptic plasticity, as a stand-alone paradigm, is necessary to maintain healthy brain function and its dysregulation in AD leads to progressive and inexorable brain organ failure. The essential role of glia and their plastic functioning in regulating synaptic plasticity in homeostatic and diseased conditions remain uncertain, though their role in CNS injury is well-documented. Glial cells are not just supporting cells in the CNS, but they are integral players in processes such as synaptic pruning, glutamate scavenging, the glutamate-glutamine cycle, gliotransmitter and neurotransmitter release, LTP/LTD, myelination, metabolic support, synaptic transmission, and learning and memory. In AD, the disrupted proteostasis can fundamentally alter the capacity of glial cells to perform their functions, having detrimental effects on neuronal function and plasticity and resulting in a feed-forward toxicity cycle.

So far, there are no effective disease-modifying therapies for AD. Many of the available drugs used in the clinic as symptomatic therapies in AD are neuroplasticity modulators with a possible role in altering neuroinflammation. More research needs to be done in terms of utilizing these manipulations to illuminate the concept of glia-mediated “plasticity of plasticity” phenomenon and its possible therapeutic contribution in neurodegenerative dementias. Clinical trials that can target both glial plasticity as well as synaptic plasticity should be encouraged, but the optimal therapeutic or combination of therapeutics has yet to be determined. As with any treatment or therapy, the optimal time of intervention is a key factor to be considered, especially while attempting to re-instate synaptic plasticity by rebalancing glial function and plasticity in a milieu of neurodegenerative proteinopathy.

## Author Contributions

PC and EK conceived the original idea and wrote the manuscript. EK drafted the manuscript and designed the tables and figures and performed PubMed. PC reviewed PubMed searches. All authors contributed to the article and approved the submitted version.

## Conflict of Interest

The authors declare that the research was conducted in the absence of any commercial or financial relationships that could be construed as a potential conflict of interest.

## Abbreviations

AAV: adeno-associated viruses
Aβ: Amyloid β
AD: Alzheimer’s disease
AICD: APP intracellular domain
AMPAR: α-amino-3-hydroxy-5-methyl-4-isoxazolepropionic acid receptor
APOE: Apolipoprotein E
APP: amyloid precursor protein
ARTAG: aging-related tau astrogliopathy
ATP: adenosine triphosphate
CBD: corticobasal degeneration
CNS: central nervous system
CSF: cerebrospinal fluid
Cx3CR1: CX3C chemokine receptor 1
C1q: complement component 1q
DBS: deep brain stimulation
DMN: default mode network
FDG-PET: fluorodeoxyglucose-positron emission tomography
FTD: frontotemporal dementia
GABA: γ-aminobutyric acid
GFAP: glial fibrillary acidic protein
LTD: long-term depression
LTP: long-term potentiation
MAPT: microtubule associated protein tau
MCI: mild cognitive impairment
NFT: neurofibrillary tangle
NMDAR: N-Methyl-d-aspartic acid or N-Methyl-d-aspartate receptor
OPC: oligodendrocyte progenitor cells
PART: primary age-related tauopathy
PET: positron emission tomography
PSP: progressive supranuclear palsy
tDCS: transcranial Direct Current Stimulation
Tg: transgenic
TMS: transcranial magnetic stimulation
TREM: triggering receptor expressed on myeloid cells.
