# Supplementary material for: Tau-Mediated Dysregulation of Neuroplasticity and Glial Plasticity
Source: Front Mol Neurosci. 2020 Aug 21;13:151. doi: 10.3389/fnmol.2020.00151 (PMC7472665; doi:10.3389/fnmol.2020.00151)
Supplement: Supplementary file 1 [file Table_1.docx]

**Supplemental Table 1: Interventions to improve synaptic plasticity in rodent models**

| **Mouse model** | **Experimental paradigm** | **Effect on neuropathology** | **Effect on synaptic plasticity** | **Effect on neuroinflammation** | **Effect on neurodegeneration** | **Reference** |
| --- | --- | --- | --- | --- | --- | --- |
| **AD mouse models** | | | | | | |
| APP/PS1 mice | Social housing | No effect | Increased synaptic function, dendritic spine density and synaptic proteins; improved performance in MWM test | Increased levels of modulators involved with regulation, growth, and inflammatory processes in astrocytes; promoted decrease of proinflammatory mediators and increase in anti-inflammatory mediators in microglia | Not reported | **(Liang et al. 2019)** |
| Aβ 1-42 injection model (C57BL/6N mice – 8 weeks old) | Intracerebroventricular injection of human Aβ 1-42; intraperitoneal injections of fisetin | Decreased Aβ accumulation, BACE-1 expression, and phosphorylation of tau at pSer413 | Increased levels of presynaptic and postsynaptic proteins; improved memory in MWM test | Suppression of gliosis | Suppressed apoptotic neurodegeneration in hippocampus | **(Ahmad et al. 2017)** |
| 5XFAD mice  PS19 mice | Auditory GENUS (gamma entrainment using sensory stimulus) | Reduced Aβ load in 5XFAD mice  Reduced phosphorylated tau in P301S mice | Improved recognition and spatial memory in NOR test | Increased astrocytosis and microgliosis; induced a clustering response by microglia | Not reported | (Martorell et al. 2019) |
| 5XFAD mice (6 mo) | JZL184 (Monoacylglycerol lipase (MAGL) inhibitor) | Reduced production and deposition of Aβ | Increased synaptic spine density; increased NMDAR and AMPAR subunits; prevents spatial learning and memory deficits | Reduced astrocytes and microglia (reduced GFAP and cd11b positive cells) | Reduced cell death in cortex and hippocampus | (Chen et al. 2012) |
| Sprague–Dawley rats injected with Aβ1-40 in the hippocampus | MDA7 (Cannabinoid type 2 agonist) | Promoted Aβ clearance | Restored LTP and improved performance in MWM | Decreased cd11b (microglial marker) and GFAP (astrocyte marker); decreased IL-1β | Not reported | (Wu et al. 2013) |
| APPNLh/NLh × PS1P264L/P264L mice (11mo) | Small molecule: MW-151 | No effect | Prevented loss of synaptic proteins (PSD95, synaptophysin, syntaxin, and SNAP25); increased LTP | Reduced IL-1β but not IL10 protein levels; less microglial and astrocytic immunoreactivity and fewer number of microglia | Not reported | (Bachstetter et al. 2012) |
| 5XFAD mice | Optogenetically driving fast-spiking parvalbumin-positive (FS-PV)-interneurons at gamma (40 Hz) | Reduced Aβ1–40 and Aβ1–42 | Synaptophysin density is unchanged | Distinct morphological change in microglia (more engulfing) | Not reported | (Iaccarino et al. 2016) |
| Intracerebroventricular Aβ25-35 injections in rats (3mo old Long-Evans rats) | Cholinergic depletion (IgG-Saporin neurotoxin that targets ChAT neurons injected into the medial septum and diagonal band of Broca) | No effect | Longer acquisition latency in MWM task | Decreased GFAP in thalamus; no change in microglial marker (OX-6) | Not reported | (Deibel et al. 2016) |
| Intracerebroventricular injection of Aβ oligomers in mice (3mo old C57BL/6 mice) | Cdk5 inhibitor (roscovitine) | N/A | Not reported | Reduced expression of Tnfa, Il1b, Il10, and Nos2; reduced TNFα and IL10 protein levels | Not reported | (Wilkaniec et al. 2018) |
| APPswe/PS1dE9 mice (6-19mo) | incretin analogue D-Ala2GIP | Reduced Aβ plaque load | Not reported | Reduced GFAP immunostaining in the cortex and hippocampus | Not reported | (Duffy and Hölscher 2013) |
| TgCRND8 and APPswe/PS1dE9 | MyD88-deficient bone marrow reconstruction | Reduced Aβ plaque burden | Better performance in Barnes Maze | Reduced Iba1 positive microglia; Reduced TNF-α and CCL-2 (TgCRND8 mice) and reduced TNF-α and IL-1β (APPswe/PS1dE9 mice); enhanced microglial recruitment to Aβ plaques | Not reported | (Hao et al. 2011) |
| **Non-AD mouse models** | | | | | | |
| Aged casp6-overexpressing mice | 1 month treatment with methylene blue | N/A | Successful induction of LTP in methylene blue treatment group | Reduced GFAP and Iba1 positive cells | Reduced caspase 6 activation | **(Zhou et al. 2019)** |
| LPS (intracerebroventricular injection) | Treatment with galantamine (cholinergic agent) 14 days prior to LPS | N/A | Reduced loss of synaptophysin and PSD-95; increased dendritic spine density and dendritic branching | Decreased GFAP and cd11b markers; decreased proinflammatory cytokines | Not reported | (Liu, et al. 2018) |
| APOE knockout mice (9-24mo old) | Voluntary running exercise paradigm | N/A | Preserved synaptophysin levels; cortical Arc expression was increased (*this was in the aged controls though – WT mice; beneficial effects of exercise did not occur in APOE KO mice) | Reduced number of Iba1 positive microglia/ monocytes in the cortex and hippocampus; reduced complement activation (*this was in the aged controls though – WT mice; beneficial effects of exercise did not occur in APOE KO mice) | Not reported | (Soto et al. 2015) |
| Streptozotozotocin (STZ)-induced rat model of AD (Sprague Dawley rats) | Treatment with Apelin-13 neuropeptide | No effect | Upregulates BDNF/TRkB pathway; improves performance in NOR and Y-maze tests; increases synaptophysin | Reduced Iba1 and GFAP mRNA and protein as well as IL-1β and TNF-α | Attenuated hippocampal cell loss | **(Luo et al. 2019)** |
