# Supplementary material for: Tau-Mediated Dysregulation of Neuroplasticity and Glial Plasticity
Source: Front Mol Neurosci. 2020 Aug 21;13:151. doi: 10.3389/fnmol.2020.00151 (PMC7472665; doi:10.3389/fnmol.2020.00151)
Supplement: Supplementary file 2 [file Table_2.docx]

**Supplemental Table 2. A selection of current therapeutics in ongoing or completed clinical trials targeting synaptic plasticity/neuroplasticity**

| **Treatment/Intervention** | **AD stage** | **Rationale** | **Primary and secondary outcome measures** | **Developed by** | **Clinical Trial Identifier/Reference** |
| --- | --- | --- | --- | --- | --- |
| **Brain stimulation** | | | | | |
| Transcranial Direct Current Stimulation (tDCS) | MCI | tDCS has potential to enhance neuroplasticity and improve cognition | fMRI, word-retrieval task | Charité University Medicine, Berlin, Germany | (Meinzer et al. 2015) |
| Repetitive Transcranial Magnetic Stimulation (rTMS) | Probable AD | (rTMS) applied to the dorso-lateral prefrontal cortex (dlPFC) | Naming performance | Centro San Giovanni di Dio Fatebenefratelli, Brescia, Italy | (Cotelli et al. 2008) |
| Repetitive Transcranial Magnetic Stimulation (TMS): specifically, Repetitive Paired Associative Stimulation (PAS) | Mild AD | PAS induces LTP in humans | PAS as assessment of neuroplasticity in DLPFC and N-back task | Centre for Addiction and Mental Health  Toronto, Ontario, Canada | NCT01847586 |
| Repetitive Transcranial Magnetic Stimulation: specifically Paired Associative Stimulation (PAS) | Probable AD | PAS induces LTP in humans | N-back Task and EEG (for Theta gamma coupling); PAS to measure plasticity in DLPFC, set of neuropsychological tests, and validate a new scale for AD | Center for Addiction and Mental Health  Toronto, Ontario, Canada | NCT02537496 |
| Deep brain stimulation | AD | Providing DBS to the fornix or Meynert nucleus (NbM) could improve cognition and brain function | ADAS-cog, MRI, FDG PET imaging, CDR-SB, ADL scale, and safety outcomes | Xuanwu Hospital, Capital Medical University,  Beijing, China | NCT03352739 |
| **Cognitive training** | | | | | |
| Computer-based cognitive training | MCI | Cognitive training can reduce cognitive difficulties and decline | Neuropsychological tests investigating verbal and visual memory | Aix-Marseille University, Marseille, France | (Herrera et al. 2012) |
| Cognitive Training | MCI | Cognitive training has been shown to improve verbal memory | RBANS memory score and fMRI | Stanford University, Stanford, CA, USA | (Rosen et al. 2011) |
| Cognitive training | Older adults (60+) | Cognitive training can lead to improvement in attention and working memory | Digit Vigilance Test, Digit Span Test, Wechsler Memory Scale | The University of Hong Kong, Hong Kong | (Leung et al. 2015) |
| Perceptual learning | Mild or moderate AD | Cortical circuits for structure-from-motion may still be plastic in mild AD | Structure-from-motion task | Keimyung University, Dalseo-Gu, Daegu, Republic of Korea | (Kim and Park 2010) |
| Cognitive training for updating and inhibition | Healthy older adults | Cognitive stimulation has a protective effect on age-related cognitive decline | Updating score, inhibition measures, Alpha-span task, Reading span task, virtutal car ride task, grey matter volume assessment, cortical thickness, updating activation, and inhibition activation | CRIUGM,  Montréal, Quebec, Canada | NCT03532113 |
| Cognitive stimulation | MCI or mild AD | Cognitive stimulation could improve cognition | MMSE, CDR, and a battery of cognitive tests | Sapienza University of Rome, Rome, Italy | NCT03784183 |
| Computerized cognitive training | MCI | Systematic cognitive training can improve cognitive performance | ADAS-Cog, UPSA, neuropsychological test score, Pfeffer functional activities questionnaire | New York State Psychiatric Institute New York, NY, USA | NCT03205709 |
| Cognitive training (Vision-based speed of processing) | MCI due to AD | Cognitive training can protect cognitive function in MCI | UFOV, EXAMINER, TIADL, fMRI, DTI, FSL | University of Rochester Memory Care Program,  Rochester, NY, USA | NCT02559063 |
| Computerized Musical Training | MCI | Musical training has a structural complexity as well as an emotional drive that may enhance neuroplastic effects | Use EEG to assess strength of cortical activity as well as change in cortical connectivity. Test audiovisual integration and assess attention, memory, and overall performance | Laboratory of Medical Physics  Thessaloníki, Greece | NCT03786185 |
| **Brain stimulation and cognitive training** | | | | | |
| Transcranial direct current stimulation (tDCS) ± working memory training | MND-AD | Working memory training could alleviate the effects of MND in older adults | N-back Task, NPI, ADAS-Cog, assessment of adverse effects | Department of Psychiatry, The Chinese University of Hong Kong | (Cheng et al. 2015) ChiCTR-TRC- 14005036 |
| Transcranial direct current stimulation and cognitive rehabilitation | MCI | Brain stimulation can enhances brain region/network functioning and increases efficacy of memory rehabilitation | fMRI and battery of cognitive tests | VA Ann Arbor Healthcare System,  Ann Arbor, MI, USA | NCT02155946 |
| **Physical activity** | | | | | |
| Resistance training | MCI | Exercise can be used to combat cognitive decline | Stroop test, Trial Making Tests, verbal digits tests, Everyday Problems Test, fMRI, Short Physical Performance Battery, and Six-Minute Walk Test | University of British Columbia, Vancouver, BC, Canada | (Nagamatsu et al. 2012) |
| Aquatic and land-based exercise | Elderly women | Exercise may improve brain function | Serum Aβ, serum HSP27, arterial PWV | Pusan National University, Busan, South Korea | (Kim et al. 2018) |
| Social dancing | Older adults at risk for dementia | Dancing could prevent or delay cognitive decline | Battery of tests assessing executive function as well as fMRI, to assess neuroplasticity while performing a series of tests | Albert Einstein College of Medicine  Bronx, New York, USA | NCT03475316 |
| Dance and Kinesthetics paired with nutrition counseling and vascular risk management | MCI | Dance intervention may improve cognitive function | ADAS-Cog, MST, VFT, NCT, DSST, TMT, MRI, GDS, NPI-Q, EQVAS, IADL, DAD, and BBS tests/scores | Institute for Neurosciences, St. Luke's Medical Center  Quezon, Metro Manila, Philippines | NCT04301544 |
| **Physical activity and cognitive training** | | | | | |
| Exergames (virtual reality-enhanced exercise) | MCI | Exercise yields cognitive benefits and exergames may increase participation | Tests to assess cognitive flexibility and simultaneous processing (Stroop, Digits Backwards, Trails Difference Score) as well as weight | Union College, New York, NY, USA | (Anderson-Hanley et al. 2012) NCT01167400 |
| Cognitive and physical training | MCI | Cognitive and physical exercise improves global cognition | CVLT, TMT, physical fitness, episodic memory, working memory, executive function, WHOQoL, IADL, GDS, and EEG/ERPs | Aristotle University Of Thessaloniki; | (Styliadis et al. 2015) NCT02313935 |
| Auditory discrimination training paired with physical fitness training | MCI or mild AD | Cognitive training and physical activity could slow or reverse cognitive decline | Battery of cognitive tests assessing memory, attention, and executive function as well as electrophysical, MRI, blood, and liquor measurements | University of Konstanz, Konstanz, Germany | NCT01061489 |
| Cognitive training and physical exercise | MCI | Cognitive training and physical activity could help prevent the progression of MCI to AD | MoCA, Stroop test, Dual taslk test, TUG test, WMS, IADL, SAIS, MAC-Q, Spatial span test, GDS-SF, CIQ, ECog | Chang Gung Memorial Hospital  Taoyuan, Taiwan | NCT03619577 |
| **Drug treatment** | | | | | |
| Liraglutide (GLP-1 analogue) | Probable AD | Liraglutide could normalize synaptic plasticity, stabilize brain glucose uptake, and reduce amyloid | Glucose metabolic rate, ADAS Exec, MRI, microglial activation, CSF markers, tau deposition, cortical amyloid, and assess adverse events | Imperial College, Hammersmith Hospital, London, United Kingdom | (Femminella et al. 2019) NCT01843075 |
| Rotigotine (dopamine D2 agonist) | Mild AD | Dopamine may modulate cortical activity in AD patients | Assessment of central cholinergic activity and cortical excitability | Tor Vergata University, Rome, Italy | (Martorana et al. 2013, Koch et al. 2014) |
| AZD0530 or saracatinib  (Src and Abl family kinases inhibitor)  (Phase 2) | Probable AD | Src family kinases are known to affect synaptic plasticity | 18F-FDG PET imaging, assessment of adverse events, ADAS-cog11, MMSE, ADCS-ADL, CDR-SO, brain volume measurement, and CSF tTau, pTau, and Aβ42 | Yale University, New Haven, CT, USA | NCT02167256 |
| PDE3I or Cilostazol  (Cyclic adenosine monophosphate phosphodiesterase 3 inhibitor)  (Phase 4) | Mild to moderate AD | PDE3I upregulates CREB which plays a role in memory and synaptic plasticity | FDG PET, ADAS-cog, MMSE, ADCS-ADL, CDR-SB, and Fazekas scale | SMG-SNU Boramae Medical Center,  Seoul, Republic of Korea | NCT01409564 |
| **Drug treatment and cognitive training** | | | | | |
| Donepezil (AChE inhibitor) and error-free word production practice  (Phase: 1) | AD | AChE inhibitors delay cognitive decline on their own. If paired with cognitive training, may improve cognition | MMSE, CVLT, BNT, WAB, REY-O, COWAT | Brain Rehabilitation Research Center, Malcom Randall VA Medical Center, Gainesville, FL, USA | (Gonzalez Rothi et al. 2009) |
| **Multimodal therapies** | | | | | |
| A multimodal lifestyle change intervention (dietary, physical activity and cognition) combined with dietary supplement epigallocatechin gallate (EGCG) | Individuals at risk for AD who are APOE4 carriers | A multimodal lifestyle change intervention plus dietary supplement with epigallocatechin gallate (EGCG) may reduce cognitive decline and improve brain connectivity in individuals with subjective cognitive decline (SCD) | ADCS-PACC-like score, fMRI, changes in structural connectivity | Barcelonabeta Brain Research Center,  Barcelona, Spain | NCT03978052 |
| Combined exercise (aerobic and resistance training), cognitive training, and vitamin D supplement | MCI | Exercise and cognitive training could improve cognition and mobility | ADASCog, CCNA cognitive battery, gait velocity, gait variability, structural MRI, fMRI, serum BDNF, Pooled Index, serum IL6, serum CRP, and serum VEGF | University of British Columbia, Vancouver, BC, Canada | (Montero-Odasso et al. 2018) NCT02808676 |
